# Differential Cytotoxicity Induced by Transition Metal Oxide Nanoparticles is a Function of Cell Killing and Suppression of Cell Proliferation

**DOI:** 10.3390/ijms21051731

**Published:** 2020-03-03

**Authors:** Larry M. Tolliver, Natalie J. Holl, Fang Yao Stephen Hou, Han-Jung Lee, Melissa H. Cambre, Yue-Wern Huang

**Affiliations:** 1 Department of Biological Sciences, Missouri University of Science and Technology, Rolla, MO 65409, USA; larry.tolliver@thermofisher.com (L.M.T.); njhcm8@mst.edu (N.J.H.); mhcxv8@mst.edu (M.H.C.); 2 Department of Biomedical Sciences, University of Wisconsin-Milwaukee, Milwaukee, WI 53211, USA; fyshou@gmail.com; 3 Department of Natural Resources and Environmental Studies, National Dong Hwa University, Hualien 97401, Taiwan; hjlee@gms.ndhu.edu.tw

**Keywords:** nanoparticle, cell proliferation, transition metal oxide, cell cycle, apoptosis

## Abstract

The application of nanoparticles (NPs) in industry is on the rise, along with the potential for human exposure. While the toxicity of microscale equivalents has been studied, nanoscale materials exhibit different properties and bodily uptake, which limits the prediction ability of microscale models. Here, we examine the cytotoxicity of seven transition metal oxide NPs in the fourth period of the periodic table of the chemical elements. We hypothesized that NP-mediated cytotoxicity is a function of cell killing and suppression of cell proliferation. To test our hypothesis, transition metal oxide NPs were tested in a human lung cancer cell model (A549). Cells were exposed to a series of concentrations of TiO_2_, Cr_2_O_3_, Mn_2_O_3_, Fe_2_O_3_, NiO, CuO, or ZnO for either 24 or 48 h. All NPs aside from Cr_2_O_3_ and Fe_2_O_3_ showed a time- and dose-dependent decrease in viability. All NPs significantly inhibited cellular proliferation. The trend of cytotoxicity was in parallel with that of proliferative inhibition. Toxicity was ranked according to severity of cellular responses, revealing a strong correlation between viability, proliferation, and apoptosis. Cell cycle alteration was observed in the most toxic NPs, which may have contributed to promoting apoptosis and suppressing cell division rate. Collectively, our data support the hypothesis that cell killing and cell proliferative inhibition are essential independent variables in NP-mediated cytotoxicity.

## 1. Introduction

Nanotoxicology is the study of nanomaterial toxicity. Nanomaterials are defined as any particulate or agglomerate that has at least one dimension in the size range from 1 to 100 nm [1]. Nanomaterials are being used with an increasing frequency in a variety of industries. Their use is common in semiconductors [2], electronics [3], pharmaceuticals [4], cosmetics [5], consumables [6], and drug delivery platforms being studied for cancer therapy [7]. It is estimated that by the year 2020 the global market for nanomaterial-based applications will reach approximately $3 trillion, and there will be six million workers in the nanotechnology sector worldwide [8,9]. With the use of nanomaterials increasing in frequency and application, exposure amongst the general public and occupational workers has become a concern. While toxicological data exists for some of the microscale equivalents of nanoparticles (NPs), the information cannot predict nanotoxicity, as nanoscale materials have different physical and chemical properties than their microscale equivalents [10]. Particles of smaller size, specifically NPs, can be inhaled deeper into the lungs than larger particles and may enter the circulatory system, resulting in inflammation, systemic distribution, cardiovascular disease, and potential neurological effects [11]. To date, there have not been any epidemiological studies or clinical evidence of NPs causing adverse health effects in humans [12]. However, studies have shown toxicity of select NPs in animal models or in vitro studies [13]. The National Institute for Occupational Safety and Health has designated workplace and occupation exposure limit recommendations for some particles based on their size. For example, TiO_2_ exposure is suggested to be limited to 2.4 mg/m^3^ for particles less than 2.5 μm and 0.3 mg/m^3^ for particles less than 0.1 μm during work days lasting up to 10 h during a 40-h work week [12]. Certain regulations on consumer exposure through food have also been established [6,14].

Our previous studies have demonstrated relationships between NPs, production of reactive oxygen species (ROS), and perturbation of intracellular Ca^2+^ concentrations ([Ca^2+^]_in_) [15,16,17]. NPs increase [Ca^2+^]_in_. The moderation of this increase is attributed to the influx of extracellular calcium, membrane integrity disruption, and perturbation of store-operated calcium entry. The increases in intracellular ROS levels may also have multiple sources. There exist synergistic relationships between [Ca^2+^]_in_ and oxidative stress (OS) as the increases in both can be reduced by antioxidants. Finally, while [Ca^2+^]_in_ and OS affect the activity of each other, they both induce cell death by distinct pathways [17]. By systematically studying seven oxides of transition metals in the fourth-period of the periodic table of elements (Ti, Cr, Mn, Fe, Ni, Cu, Zn), we delineated that cytotoxicity is a function of particle surface charge, relative number of particle surface binding sites, and metal ion dissolution rate [18].

NP-mediated toxicity is a rather complicated process and more factors, other than the ones we have determined, are at work. There have been numerous reports on titanium oxide [19], nanogold [20], carbon nanotubes [21], silica oxide [22], aluminum oxide [15], and cerium oxide [23] relating to cell killing. The final cell number that researchers observed was more than just the effect of cell death. Indeed, the outcome of reduced cell number could be the consequence of cell proliferation rate alteration. Herein, we hypothesize that cell viability is a function of cell killing and suppression of cell proliferation. To test our hypothesis, we conducted time- and dose-dependent cytotoxicity studies using human bronchoalveolar carcinoma (A549) cells as a model. Particle properties such as shape, size, and specific surface area were characterized by transmission electron microscopy (TEM) and the Brunauer–Emmett–Teller (BET) method. Apoptosis measurement was performed with flow cytometry and verified with cellular imaging. Tritiated thymidine incorporation was used to determine the rate of cell proliferation. Toxicity was ranked according to severity of cellular responses and a correlation analysis between viability, proliferation, and apoptosis was conducted to visualize the strength of relation between the toxic responses induced by each NP. Alteration of cell cycle was assessed to ascertain whether it contributed to changes in cell proliferation in the two most toxic NPs.

## 2. Results

### 2.1. Characterization of Nanoparticles

Physical properties of the seven transition metal oxide NPs were previously characterized (Table S1) [18]. The approximate physical sizes (APS) of the NPs ranged from 16 ± 5 nm (NiO) to 82 ± 31 nm (Mn_2_O_3_). The APS measured in the report were similar to those in the data sheet provided by the manufacture. TEM revealed needle-like (TiO_2_), nearly spherical (Cr_2_O_3_, NiO, CuO, ZnO), or spherical (Mn_2_O_3_, Fe_2_O_3_) morphology for each of the NPs. The lowest specific surface area (SSA) of the NPs was 8.7 m^2^/g (Mn_2_O_3_) and SSA ranged to a high of 179 m^2^/g (TiO_2_). It was noted that while TiO_2_, Fe_2_O_3_, and CuO had similar APS, they possessed distinctly different SSA. This could be due to differences in surface porosity, morphology, or particle aggregation.

### 2.2. Reduction of Cytotoxicity

**Figure 1:**
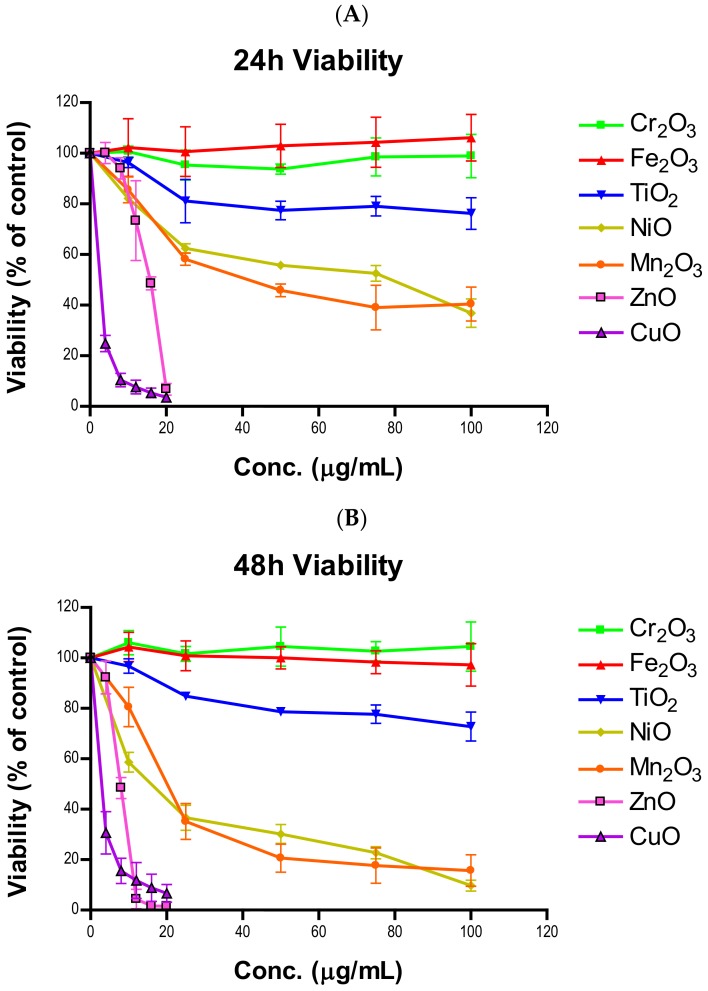
Viability of A549 cells after (**A**) 24- or (**B**) 48-h exposure to various concentrations of one of seven nanoparticles.

Concentration- and time-dependent cytotoxicity of the seven transition metal oxides is summarized in Figure 1. These NPs can be arranged in three cytotoxicity “tiers” with Fe_2_O_3_, Cr_2_O_3_ and TiO_2_ being nontoxic to mildly toxic, NiO and Mn_2_O_3_ being moderately toxic, and ZnO and CuO being highly toxic. The two highly toxic particles were so devastating that the concentration ranges for these particles had to be lowered to 0–20 µg/mL from 0–100 µg/mL that was used for all other particles. Specific viability percentages can be found in Supplementary Table S2 (24-h) and Supplementary Table S3 (48-h). In the low toxicity group, Fe_2_O_3_ and Cr_2_O_3_ did not produce notable changes in viability (*n* = 3, *p*’s > 0.05). The 24- and 48-h viabilities of 100 µg/mL of TiO_2_ were similar, being 76.2 ± 5.1% at 24 h and 72.8 ± 4.7% at 48 h. A significant decrease in viability upon TiO_2_ exposure was observed at both time points and all concentrations except for 24-h 10 µg/mL (*n* = 3, *p*’s < 0.05). Both moderately toxic particles (NiO and Mn_2_O_3_) demonstrated greater viability decline in the 48-h group compared to the 24-h group and had significant decrease in viability at all times and concentrations (*n* = 3, *p*’s < 0.05). The 100 µg/mL of NiO dose resulted in 36.8 ± 4.6% at 24 h and 9.7 ± 1.8% at 48 h, with the 100 µg/mL of Mn_2_O_3_ producing similar changes of 40.4 ± 5.5% at 24 h and 15.7 ± 5.1% at 48 h. In the high toxicity group, greater toxicity was also observed with ZnO at 48 h. ZnO produced significant changes at 16 and 20 µg/mL after 24 h and all concentrations except 4 µg/mL after 48 h (*n* = 3, *p*’s < 0.05). The responses varied between concentrations for ZnO with the values at 8 µg/mL being 94.0 ± 3.4% for 24 h but 48.4 ± 3.4% for 48 h. By doubling the concentration, the viability at 16 µg/mL dropped to 48.6 ± 2.1% for 24 h and 1.6 ± 0.1% for 48 h. At 20 µg/mL, almost no living cells were detected. CuO viabilities were similar at both 24 and 48 h across all concentrations but were all significantly different from the controls (*n* = 3, *p*’s < 0.05). CuO exposure of 20 µg/mL resulted in a viability of 3.5 ± 1.0% at 24 h and 6.6 ± 2.9% at 48 h. The toxicity trend is similar to that of our previous findings [18]. This indicates that 1) the NPs have been stable in the storage condition specified in the Materials and Methods and 2) variabilities between multiple experimenters are negligible.

### 2.3. Induction of Apoptosis

**Figure 2:**
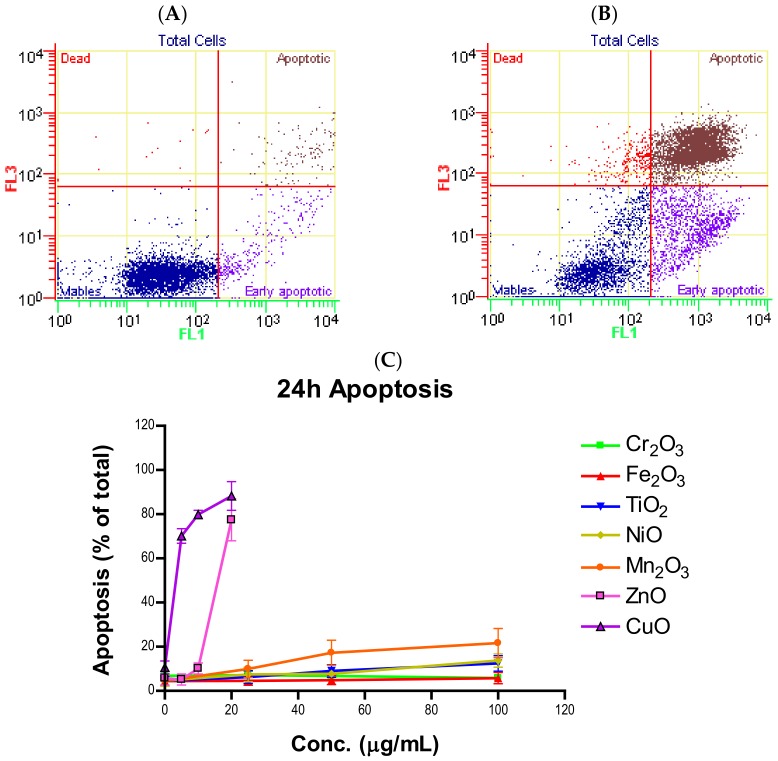

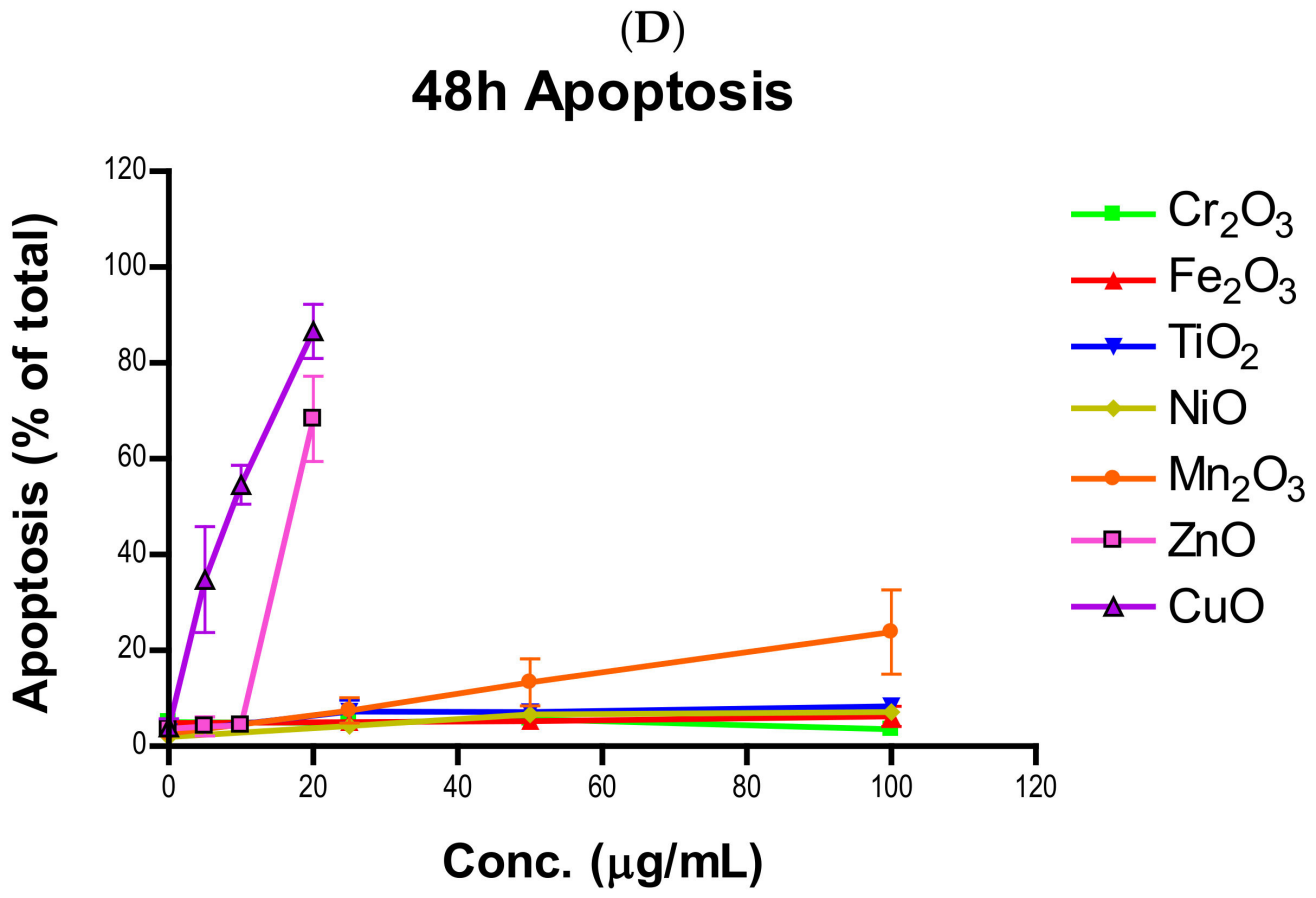
Flow cytometry gating of cells treated with (**A**) 0 and (**B**) 20 μg/mL of ZnO after 24 h. Total apoptosis of A549 cells after (**C**) 24- or (**D**) 48-h exposure to various concentrations of one of seven nanoparticles.

We combined early apoptotic cells with late apoptotic cells to determine the total proportion of cells undergoing apoptosis at each time point. The total apoptotic percentage in the treatment groups was compared to the corresponding control groups. The total apoptotic percentages for all seven NPs at 24 and 48 h are summarized in Figure 2 (Supplementary Tables S4 and S5). For the low to mildly toxic tier, both time points and all concentrations of Cr_2_O_3_ and Fe_2_O_3_ showed no difference in apoptosis when compared to the control group (*n* = 3, *p*’s > 0.05). TiO_2_ at 24 h exhibited elevated apoptosis at 100 µg/mL (12.5 ± 2.9%) and at all concentrations at 48 h (*n* = 3, *p*’s > 0.05). For the moderate tier, NiO had significant apoptosis at 100 µg/mL (13.8 ± 2.5%) at 24 h and Mn_2_O_3_ induced significant apoptosis at 50 µg/mL (17.3 ± 4.6%) as well as at 100 µg/mL (21.6 ± 5.4%) (*n* = 3, *p*’s < 0.05). NiO at 48 h exhibited significant apoptosis induction at all concentrations tested while Mn_2_O_3_ only displayed significant increase in apoptosis at 100 µg/mL (23.8 ± 7.2%) compared to the control (*n* = 3, *p*’s < 0.05). In the high toxicity tier, ZnO had significant induction at only 20 µg/mL at both 24-h (77.5 ± 7.8%) and 48-h (68.4 ± 6.3%) time points (*n* = 3, *p*’s < 0.05). All tested doses of CuO produced significant apoptosis in both time groups, with 100 µg/mL causing apoptosis in extremely high numbers at 24 (88.2 ± 5.3%) and 48 h (86.6 ± 4.6%) (*n* = 3, *p*’s< 0.05).

### 2.4. Alteration of Cell Morphology

**Figure 3:**
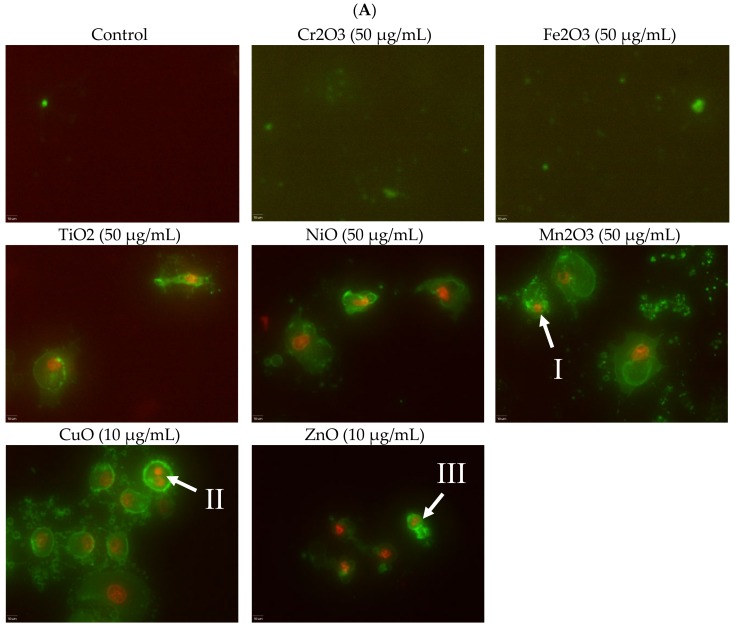

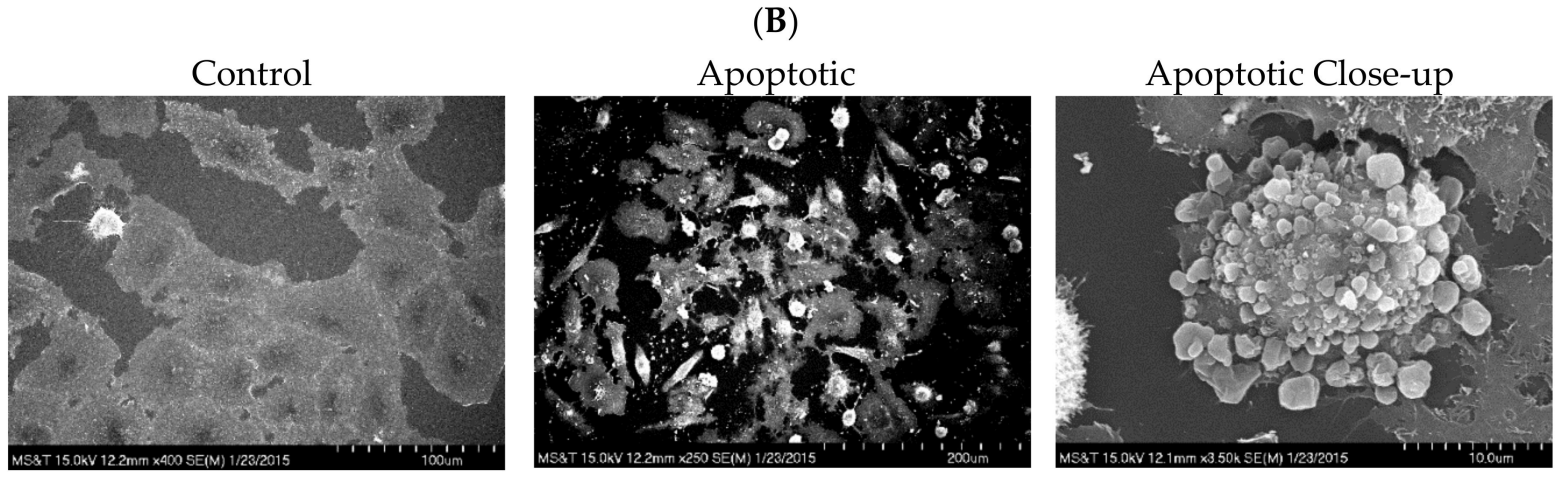
(**A**) Fluorescence apoptotic stains with Annexin V-FITC and 7-aminoactinomycin D (7-AAD) after cells were exposed to nanoparticles at 50 or 10 µg/mL. Green color alone indicates cells undergoing early apoptosis. Red and green in combination indicate cells undergoing late apoptosis. Examples of (I) blebbing, (II) nuclear fragmentation, and (III) apoptotic bodies are marked. Scale bar is 10 μm. (**B**) SEM images of A549 cells after exposure to 50 μg/mL MnO. Membrane blebbing is quite noticeable in the close-up image.

Alterations in cell morphology due to NP exposure were observed both by epifluorescence microscopy (Figure 3A) and scanning electron microscopy (SEM) (Figure 3B). Apoptotic morphologies such as membrane blebbing, nuclear fragmentation, and apoptotic bodies were clearly observed. Membrane blebbing was particularly noticeable under the SEM close-up image. The severity of apoptosis observed was similar to the trend set by viability results, with Cr_2_O_3_ and Fe_2_O_3_ treatment resulting in none to minimal toxicity and small numbers of apoptotic cells, Mn_2_O_3_ and NiO having moderate numbers, and ZnO and CuO having high numbers, with almost no healthy cells remaining.

### 2.5. Suppression of Cell Proliferation

**Figure 4:**
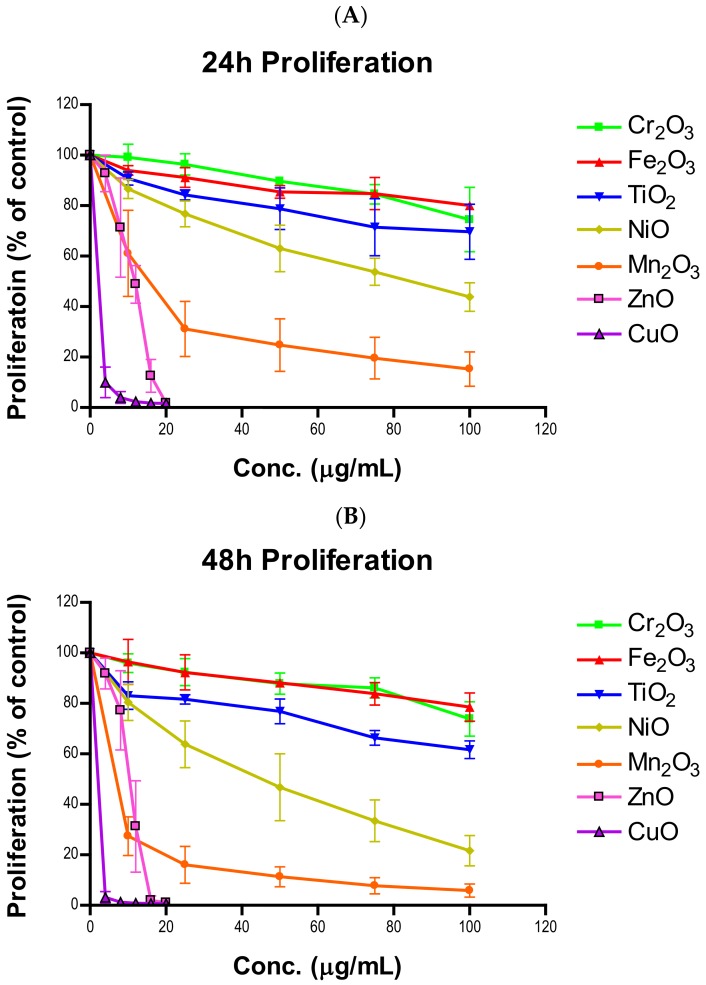
Proliferation of A549 cells after (**A**) 24-h or (**B**) 48-h exposure to various concentrations of one of seven nanoparticles.

Tritiated thymidine incorporation is indicative of cell proliferation rate. The percentage of cells proliferating was calculated by taking the average of all radioactive counts in a dosage group and dividing it by the average of the radioactive counts of the untreated group. Figure 4 shows time- and concentration-dependent suppression of cell proliferation. Significant inhibition of proliferation was observed at high doses in all seven NPs for both time points (Supplementary Tables S6 and S7). Cr_2_O_3_ and Fe_2_O_3_ treated cells were the least affected with 24-h 100 µg/mL doses having cells actively proliferating at 74.5 ± 10.4% and 79.6 ± 0.2%, respectively. These values were similar to the 48-h values, with 73.9 ± 5.6% for Cr_2_O_3_ and 75.3 ± 0.3% for Fe_2_O_3_. Although, the decreases for Cr_2_O_3_ and Fe_2_O_3_ were significant at 50, 75, and 100 µg/mL at both time points, as well as at 10 and 25 µg/mL of Fe_2_O_3_ after 24 h (*n* = 4, *p*’s < 0.05). TiO_2_ differed from the rest of the low toxicity group by having greater decreased proliferative percentages at 100 µg/mL (69.6 ± 8.9% for 24 h and 61.6 ± 2.9% for 48 h), with significant decreases observed at all concentrations and times (*n* = 4, *p*’s < 0.05). The moderately toxic group also produced a significant decrease at all times and concentrations (*n* = 4, *p*’s < 0.05). While NiO had high time-dependent proliferative reduction at 100 µg/mL (43.8 ± 4.6% for 24 h and 21.6 ± 4.9% for 48 h), Mn_2_O_3_ inhibition of proliferation at 24 (15.2 ± 5.5%) and 48 h (5.8% ± 2.2%) was much less prominent. ZnO had complete inhibition of proliferation at both 24 (1.7 ± 0.3%) and 48 h (1.0 ± 0.7%) at 20 µg/mL, which should be considered background level radiation. ZnO doses of 12, 16, and 20 µg/mL exhibited significantly decreased proliferation at both time points (*n* = 4, *p*’s < 0.05). Cells exposed to 20 µg/mL of CuO experienced a similar degree of inhibition at both 24 (1.7 ± 0.6%) and 48 h (0.6 ± 0.3%), though all times and concentrations were significantly decreased (*n* = 4, *p*’s < 0.05).

### 2.6. Alteration of Cell Cycle

**Figure 5:**
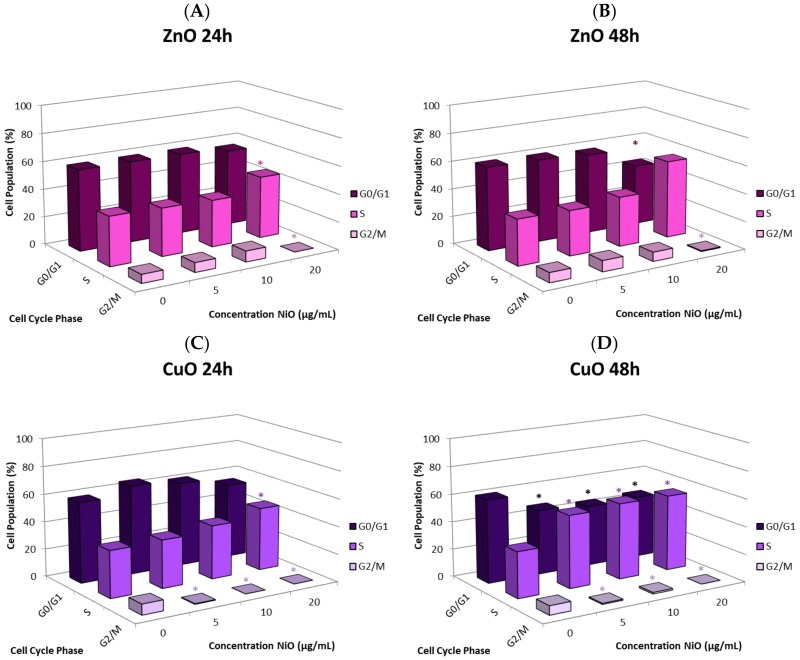
Alteration of cell cycle of A549 cells after exposure to various concentrations of ZnO NPs for (**A**) 24 h and (**B**) 48 h or CuO NPs for (**C**) 24 h or (**D**) 48 h. Values significantly different from the control (*p*’s < 0.05) are indicated with *.

We selected ZnO and CuO to study the alteration of the cell cycle (Figure 5, Supplementary Figure S1, and Table S8). An increase in the S phase and a decrease in the G_2_/M phase at 20 µg/mL of ZnO were observed in both 24 and 48 h (*n* = 3, *p*’s < 0.05). Compared to 24 h (+9.3%), the increase in the S phase was more prominent at 48 h (+17.1%), which might have contributed to the more significant decrease in G_0_/G_1_ (−11.0%, *n* = 3, *p*’s < 0.05). Similar patterns occurred upon exposure to CuO (*n* = 3, *p*’s < 0.05). However, CuO exhibited significant differences from the control at lower concentrations, with almost no cells observed in G_2_/M at any concentration. At 24 h, S phase increased significantly at 20 µg/mL (+11.6%) and G_2_/M decreased at all concentrations of CuO, with 20 µg/mL having a change of −7.7% (*n* = 3, *p*’s < 0.05). All phases exhibited significant differences compared to the controls after 48-h CuO exposure, with 20 µg/mL producing changes in G_0_/G_1_, S, and G_2_/M of −15.2%, +21.7%, and −6.6%, respectively (*n* = 3, *p*’s < 0.05).

### 2.7. Correlation

**Figure 6:**
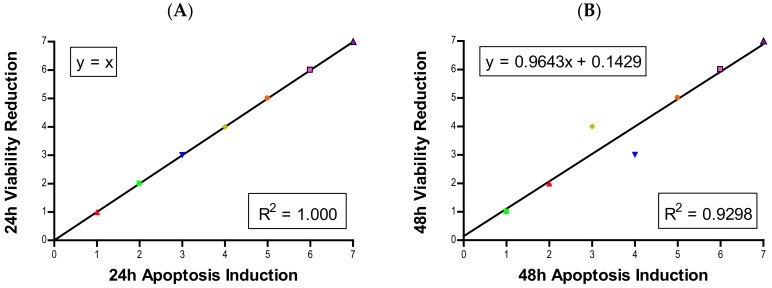

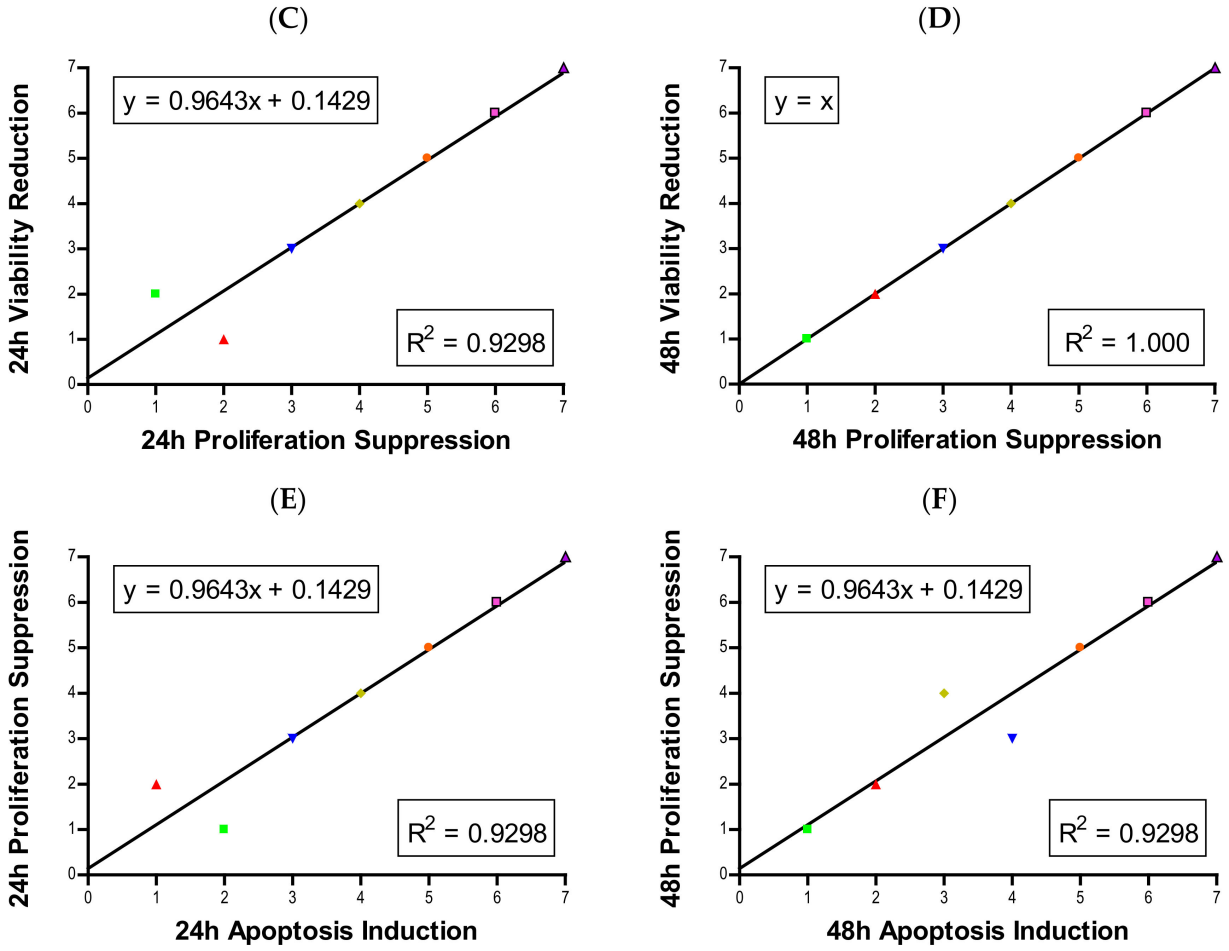
Linear regression analysis of (**A**) 24 h viability vs. proliferation (**B**) 48 h viability vs. proliferation (**C**) 24 h viability vs. apoptosis (**D**) 48 h viability vs. apoptosis (**E**) 24 h proliferation vs. apoptosis (**F**) 48 h proliferation vs. apoptosis.

The results of linear regression analyses between 24- and 48-h viability, apoptosis, and proliferation rankings are illustrated in Figure 6.

## 3. Discussion

In this study, we investigated the cytotoxicity of seven fourth-period transition metal oxide NPs and explored several cellular responses as components of cytotoxicity. We hypothesized that cytotoxicity is a function of cell killing and suppression of cell proliferation and, as such, we assayed for cell viability, apoptosis, cellular proliferation, and cell cycle progression. Data from cell viability indicated the response to NPs is concentration- and time-dependent. Importantly, data revealed that NPs exert a higher degree of toxicity towards 48-h and longer exposure. To our knowledge, the temporal stability of NPs in storage has not been addressed. We took the opportunity to compare our current 24-h viability data to the data from our previous study, which was conducted by a different experimenter [18]. Concentration-dependent patterns from both studies resembled each other, with some slight variations. This is indicative of particle stability in storage conditions. However, we did notice that TiO_2_ seemed to be more toxic in these sets of experiments (approximately 96% in previous vs. 76% in current). Due to this discrepancy, multiple researchers were asked to perform the same experiment to ensure our results were reliable, which yielded almost the same outcome. We are unsure of what caused the difference, but particle-specific aging is speculated.

Induction of apoptosis is a hallmark of NP toxicity [24]. For our purposes, early and late apoptotic events were combined into a total apoptotic value. Concentration- and time-dependent apoptotic effects were observed in TiO_2_, NiO, Mn_2_O_3_, CuO, and ZnO, with the last two showing a much more severe degree of programmed cell death. Epifluorescent microscopy and SEM images confirmed apoptotic morphologies, such as membrane blebbing and nuclear fragmentation, after exposure to NPs. Cell viability reduction correlated with apoptotic events, indicating a close pathway-dependent relationship (Figure 6A,B).

Plenty of nanotoxicological studies have focused on NP-imposed cell killing [25,26,27]. We believe that cell killing may not be the only reason that results in cell number reduction. The suppression of cell proliferation can have the same effect as cell killing in driving down cell numbers. Whether transition metal oxides have influences over cell proliferative inhibition has not been systematically investigated. In this study, all seven oxides of transition metal NPs in the fourth period of the periodic table showed time- and concentration-dependent proliferative inhibition in A549 cells. In general, proliferative inhibition followed the same tier trend as cell viability. NiO and Mn_2_O_3_ exhibited more prominent time-dependent inhibition than TiO_2_, Cr_2_O_3_, and Fe_2_O_3_. Strikingly, the degrees of time-dependent suppression of proliferation by CuO and ZnO were much steeper at 12 and 16 µg/mL as time progressed towards 48 h. A significant correlation between cell viability reduction and suppression of cell proliferation suggests a proliferative inhibition is an independent variable influencing cell number, in addition to cell killing (Figure 6C,D). Previously we developed a model to estimate the number of cells in the second generation [28]:

*Cell # in Generation 2 = 2 (Proliferating cells) + Non-proliferating cells – Dead cells (via killing)* (1)

This model assumes the doubling time of a cell line is 24 h and the rate of doubling time is not altered by the NPs. Future studies should investigate the differential contribution of these two components to the change in cell number.

The cause of the observed cell proliferation suppression may be multiple. Alteration of cell cycle induced by the NPs could have a major influence. We selected CuO and ZnO to further investigate this issue. Time- and concentration-dependent effects on cell cycle in response to NP exposure were observed. In general, there was an increase in the proportion of cells in S phase and a decrease in the G_0_/G_1_ and G_2_/M phases. Cells arresting in certain phases of cell cycle either attempt to fix damage or accumulate too much damage and undergo apoptosis. The increase in S phase indicates that cells exposed to CuO and ZnO are being stalled in S phase; the NPs may directly damage DNA or be influencing DNA replication machinery. In this study, significant correlation between suppression of proliferation and apoptosis may suggest cell cycle alteration-mediated cell death plays a role in proliferative inhibition (Figure 6E,F). Other studies also have observed cell cycle alteration induced by NPs and the phenomenon is quite dynamic. Arrest can occur in any phase, or in multiple phases of the cell cycle. Phase-specific arrest depends on cell line, particle, and particle concentration of [28,29,30,31,32,33]. For instance, in one study [34] exposure to NiO NPs resulted in a significant decrease in G_0_/G_1_ and an increase in G_2_/M in A549 cells. In contrast, exposure to NiO NPs led to a significant increase in G_0_/G_1_ and a decrease in G_2_/M in BEAS-2B cells [34]. Exposure to NiO NPs caused BEAS-2B cells arrest in the G_2_/M phase, while ZnO and Fe_2_O_3_ did not affect the cell cycle [34,35]. It is important to be aware of the fact that NPs composed of the same elements may have quite different physical and chemical properties, such as surface charge, surface area, dissolution of ions, morphology, and crystalline structure, in different studies. Consequentially, interpretations of the dynamic outcome in cell cycle alteration becomes a complicated issue if NPs are not well characterized.

## 4. Materials and Methods

### 4.1. Material Sources

A549 cells were obtained from the American Type Culture Collection (ATCC CCL-185, Manassas, VA, USA). The seven transition metal oxide NPs (TiO_2_, Cr_2_O_3_, Mn_2_O_3_, Fe_2_O_3_, NiO, CuO, and ZnO) were purchased from Nanostructured and Amorphous Materials (Houston, TX, USA). Sulforhodamine B (SRB) dye was procured from Biotium (Freemont, CA, USA). Annexin V-FITC and 7-AAD were obtained from BD Biosciences (Franklin Lakes, NJ, USA). Tritiated thymidine came from Perkin-Elmer (Waltham, MA, USA) and propidium iodide (PI) was purchased from Fisher Scientific (Pittsburgh, PA, USA).

### 4.2. Storage and Characterization of Nanoparticles

NPs were stored in an amber desiccator under a pure nitrogen atmosphere to protect them from moisture, oxidation, and UV damage. Before being used, the NPs were further dried in an oven. Characterization of the NPs followed our previous publication [18]. The shape and APS of NPs were determined by TEM. The BET method was used to measure the SSA of the NPs.

### 4.3. Cell Culture

A549 are a common in vitro cell line model for nanotoxicity testing. Cells were maintained in 10 cm tissue culture dishes at 37 °C in a 5% CO_2_ humidified incubator. The growth media was Ham’s F-12 modified medium (Corning Inc., Corning, NY, USA) supplemented with 10% HyClone FetalClone serum (GE Healthcare Life Sciences, Marlborough, MA, USA) and 1% of a combination of penicillin/streptomycin antibiotics (MP Biomedicals, Irvine, CA, USA). Cells were allowed to grow to a confluence of approximately 70–80% before being passaged or seeded for experiments. Cells were only grown for approximately 20 passages before a new vial of cells was brought up from liquid nitrogen storage.

### 4.4. Nanoparticle Treatment

NPs were prepared as a one mg per mL working solution by weighing out particles on an analytical balance and suspending them in a corresponding amount of cell culture medium. The suspensions were sonicated in sealed polyethylene vials for three minutes to break up aggregates and ensure an even mixture of NPs. NP suspensions were immediately used for experiments following preparation and were diluted to the desired concentrations in experimental dishes. For mildly and moderately toxic NPs (TiO_2_, Cr_2_O_3_, Mn_2_O_3_, Fe_2_O_3_, and NiO), 0, 10, 25, 50, 75, and 100 µg/mL were used while cells were only exposed to 0, 4, 8, 12, 16 and 20 µg/mL of highly toxic particles (CuO and ZnO). Apoptosis and cell cycle experiments were limited to 4 doses of NPs, being 0, 25, 50, and 100 µg/mL for mildly and moderately toxic and 0, 5, 10, and 20 µg/mL for highly toxic particles. Untreated cells were used as a negative control in all experiments.

### 4.5. Cell Viability Assay

A549 cells were seeded into 24-well plates at a density of 45,000 cells per well for 24-h exposure and 22,000 cells per well for 48-h exposure. Cells were treated with 0, 10, 25, 50, 75 and 100 µg/mL or 0, 4, 8, 12, 16 and 20 µg/mL for mildly and moderately or highly toxic NPs, respectively. At the end of cell exposure (24 or 48 h) to NP suspensions, the medium was discarded and the SRB assay was used to determine cell viability relative to the control group, with untreated cells being considered 100% viable [36]. Briefly, the cells were fixed with cold 10% trichloroacetic acid (TCA) for 1 h at 4 °C. The TCA solution was then discarded, and the fixed cells were washed three times with distilled water, followed by complete drying. SRB solution (0.2% in 1% acetic acid) was added to stain the cells for 30 min at room temperature. The solution containing stain was pipetted off and excess dye was eliminated from the cells by rinsing three times with 1% acetic acid. Sample wells were allowed to dry before dissociating the dye in cold 10-mM Tris buffer (pH 10.5). Stained sample solutions were transferred onto a 96-well plate in aliquots of 100 μL and absorbance was read at 510 nm using a microplate reader (FLUORstar Omega, BMG Labtechnologies, Cary, NC, USA).

### 4.6. Apoptosis Analysis

The quantification of apoptosis due to NP exposure was measured using the fluorescent dyes Annexin V-FITC and 7-AAD on a Cell Lab Quanta SC MPL flow cytometer (Beckman-Coulter, Brea, CA, USA). Annexin V-FITC binds to and labels phosphatidylserine (PS), a cell membrane phospholipid that flips to the extracellular surface during apoptosis. 7-AAD is a DNA stain that is only able to penetrate the permeable membranes of dying cells. A549 cells were allowed to grow for 24 h in 6 cm tissue culture dishes and then treated with varying concentrations of transition metal oxide NPs for 24 and 48 h. The seeding densities were 250,000 cells per dish and 120,000 cells per dish for 24- and 48-h treatments, respectively. The range of NP concentrations tested included 0, 25, 50, and 100 µg/mL for TiO_2_, Cr_2_O_3_, Fe_2_O_3_, NiO, and Mn_2_O_3_ and 0, 5, 10, and 20 µg/mL for ZnO and CuO. At the end of the exposure period, the media was removed, and the dishes were washed with phosphate buffered saline (PBS). The cells were harvested using 0.25% trypsin-EDTA (Gibco, Life Technologies, Carlsbad, CA, USA) and transferred to a centrifuge tube. The tubes were centrifuged, the supernatant was discarded, and 1 mL of ice-cold PBS was used to wash the pellet. The tubes were then centrifuged again, and PBS wash was removed. Each sample was resuspended in 5 µL of Annexin V-FITC, 5 µL of 7-AAD, and 100 µL of 1x Annexin V binding buffer (BD Biosciences, Franklin Lakes, NJ, USA). These tubes were incubated in the dark for 15 min. After incubation, another 400 µL of Annexin V binding buffer was added to each sample and 250 µL of this cell suspension was transferred to a 96-well plate (Corning Inc., Corning, NY, USA) for analysis via flow cytometry. Operating conditions for the flow cytometer were the stock apoptosis protocol included with the software. The data was exported to Microsoft Excel 2016 using the Quanta SC Analysis software and calculations of averages and standard deviations were performed in Excel. The total fraction of apoptotic cells was determined by summing the populations in early and late apoptosis.

### 4.7. Scanning Electron Microscopy Imaging

Microscopic examination of apoptotic and control cells was performed using a Hitachi S-4700 SEM (Hitachi, Tokyo, Japan). Cells were grown for 24 h at an initial density of 15,000 cells per dish in 35 mm glass bottom tissue culture dishes (MatTek, Ashland, MA, USA) before treatment with 50 μg/mL of mildly or moderately toxic NPs or 10 μg/mL of highly toxic NPs for 24 h. At the end of the exposure period, the cell culture media was removed. The dish was washed with PBS and the cells were fixed in a solution of 3% glutaraldehyde in PBS overnight. After fixation, the cells were dehydrated with an increasing series of ethanol concentrations from 50% to 100% for 15 min at a time, with the final concentration (100%) repeated once. The cells were then dried using a mixture of ethanol and hexamethyldisilazane (HMDS, Acros Organics, Fisher Scientific, Pittsburgh, PA, USA) for 15 min at a time using a ratio of 1:2 HMDS to ethanol, then 2:1, and finally ending with pure HMDS. Like the ethanol, the final HMDS step was repeated once. The dishes were then mounted on pin stubs with carbon dot adhesives or carbon paint. Then the dishes were coated with a mixture of gold and palladium for 1 min in a Hummer VI sputter coater (Anatech, Sparks, NV, USA) to provide contrast and prevent charging of the sample once in the SEM. The plastic sides of the culture dish were then pried off, leaving only the cell-containing glass cover slip on the pin stub. A piece of copper tape was run from the pin stub to the Au/Pd coated slip to provide a path to ground for the electron beam. The mounted sample was then placed into the scanning electron microscope for imaging. Operating conditions for the SEM followed the protocol of the Missouri S&T Advanced Materials Characterization Laboratory.

### 4.8. Epifluorescence Microscopy

Qualitative imaging of apoptosis induced by exposure to NPs was observed using the fluorescent dyes Annexin V-FITC and 7-AAD on an Olympus IX51 inverted epifluorescence microscope (Olympus Corporation, Tokyo, Japan). Cells were seeded on 35 mm glass bottom microscopy tissue culture dishes at 15,000 cells per dish and allowed to grow to approximately 70% confluence, around 24 h. Cells were then treated with 50 μg/mL of mildly or moderately toxic NPs or 10 μg/mL of highly toxic NPs for 24 h. Following incubation with NPs, the cell culture medium was removed, and the dishes were washed with PBS. Annexin V binding buffer mixed with 5 µL of Annexin V-FITC and 5 µL of 7-AAD was added and the dishes were incubated in the dark for 15 min. After incubation, the plates were washed with Annexin V binding buffer twice and enough buffer to cover the bottom of the dish was left on the cells to prevent drying. The plates were placed into an opaque container to keep them out of the light before imaging. The cells were imaged using the Olympus microscope, using green and red filters for Annexin V-FITC and 7-AAD, respectively.

### 4.9. Tritiated Thymidine Incorporation Assay

The tritiated thymidine ([5′-^3^H]-thymidine) incorporation assay has been widely used to study cell proliferation [37] and was used to determine the proliferation of NP-treated cells. A549 cells were plated into 24-well tissue culture plates with seeding densities of 45,000 and 22,000 cells per well for 24- and 48-h treatment periods, respectively. Cells were exposed to 0, 10, 25, 50, 75, and 100 µg/mL or 0, 4, 8, 12, 16, and 20 µg/mL for mildly and moderately or highly toxic transition metal oxide NPs, respectively. At the same time as the cells were dosed with NPs, they were also treated with 20 µL tritiated thymidine. Thymidine working solution was prepared in 500 µL of PBS with 20 µL of [5′-^3^H]-thymidine (1 µCi/µL). After 24 or 48 h of exposure, the cell culture medium was removed, and the wells were washed with ice-cold PBS twice. Following the PBS wash, the cells were quickly fixed in ice-cold 10% TCA for 5 min on ice. The TCA fixation was repeated once. After fixation, the cells were lysed using 0.5 mL of room-temperature 1 N NaOH. The same volume of 1 N HCl was used to neutralize the cell solution. The lysed cell solution was thoroughly mixed by pipetting up and down and transferred to liquid scintillation counting vials with 4 mL of Econo-Safe scintillation counting fluid (Research Products International, Mt Prospect, IL, USA). Sample vials were capped, labeled and racked for analysis in a Beckman liquid scintillation counter LS6500 (Beckman-Coulter, Brea, CA, USA). Untreated cells were considered to have 100% possible proliferative potential and treatment groups were presented as relative to the control. All radioactive waste was disposed of following Missouri S&T’s Department of Environmental Health and Safety procedures.

### 4.10. Cell Cycle Analysis

The alteration of the cell cycle due to NP exposure was measured using PI staining and subsequent analysis via flow cytometry. A549 cells were seeded into 6 cm tissue culture dishes with initial densities of 250,000 cells per dish for 24-h treatment and 120,000 cells per dish for 48-h treatment. Cells were exposed to varying concentrations of transition metal oxide NPs (0, 25, 50, and 100 µg/mL for mildly and moderately toxic and 0, 5, 10, and 20 µg/mL for highly toxic NPs) for 24- or 48-h periods. After incubation with NPs, the cells were washed, harvested using trypsin, and centrifuged down into a pellet. The cell pellet was then resuspended in 1 mL of PBS and 3 mL of ice-cold absolute methanol was added dropwise to samples being stirred with a vortex. The cells suspensions were left at least overnight in 75% methanol to ensure complete fixation. After fixation, the cells were washed once with PBS and centrifuged. The cells were then suspended in PI staining solution (50 µg/mL PI, 0.1% RNase A, and 0.05% Triton X-100 in PBS) and incubated in the dark for 20 min. RNase A was included to destroy any RNA present, as PI can also bind to RNA, and to ensure PI staining was limited to DNA. Triton X-100 ensured cell membranes were permeable to PI. After incubation, 1 mL of PBS was added to each sample. Samples were centrifuged, supernatant was removed, and pellet was resuspended in 250 mL of PBS. The stained samples were transferred into a 96-well plate and analyzed with a CytoFLEX flow cytometer (Beckman-Coulter, Brea, CA, USA). Alterations of cell cycle due to NP exposure were determined through PI fluorescent intensity, as different phases of the cell cycle have differing amounts of DNA content. The percentage distribution of cells in each phase of the cell cycle (G_0_/G_1_, S, and G_2_/M) was determined using FCS Express 6 (DeNovo software, Pasadena, CA, USA).

### 4.11. Statistical Analysis

Each experiment was repeated at least three times independently with treatment groups having multiple samples. Data are presented as mean ± standard deviation. Statistical analysis was completed in Minitab 19. One-tailed unpaired t-tests were used to compare experimental groups to the control group in normalized data sets, with µ > control or µ < control depending on the experimental hypothesis. Analysis of variance (ANOVA) with Dunnett comparison was used to determine values statistically significant from control groups. Significance was set at *p* < 0.05. The majority of figures were produced using GraphPad Prism 4 except for cell cycle distribution graphs, which were produced by Microsoft Excel 2016. For correlation analysis, each NP in each assay was assigned a rank from 1 to 7, with 1 having the least effect and 7 having the most severe effect. Once the particles were ranked for every assay, ranks were compared against one another using linear regression analysis in GraphPad Prism 4. The R^2^-value was calculated and displayed on each plot. A high R^2^ value indicates strong correlation while a low R^2^ value is indicative of little-to-no correlation.

## 5. Conclusions

Our data support the hypothesis that NP-imposed cytotoxicity is a function of cell killing and cell proliferative inhibition. The adverse effects are time and concentration dependent. Compared to our study in 2013, cytotoxicity pattern is comparable indicating NPs have been stable in our storage conditions over the years. Apoptosis and signature morphological changes were confirmed via flow cytometry, fluorescent microscopy, and SEM observation. Cell arrest in the cell cycle leading to apoptosis may play a role in suppressing cell division rate.

## Supplementary Materials

Supplementary materials can be found at https://www.mdpi.com/1422-0067/21/5/1731/s1, or by contacting the corresponding author.

## Abbreviations

NP	Nanoparticles
OS	Oxidative Stress
TEM	Transmission electron microscopy
SEM	Scanning electron microscopy
BET	Brunauer-Emmett-Teller
APS	Approximate physical size
7-AAD	7-aminoactinomycin D
