# Supplementary material for: Differential Cytotoxicity Induced by Transition Metal Oxide Nanoparticles is a Function of Cell Killing and Suppression of Cell Proliferation
Source: Int J Mol Sci. 2020 Mar 3;21(5):1731. doi: 10.3390/ijms21051731 (PMC7084189; doi:10.3390/ijms21051731)
Supplement: Supplementary file 1 [file ijms-21-01731-s001.pdf]

International Journal of *Molecular Sciences*

Supplementary Materials

Differential Cytotoxicity Induced by Transition Metal Oxide Nanoparticles is a Function of Cell Killing and Suppression of Cell Proliferation

Larry M. Tolliver [1], Natalie J. Holl [1], Fang Yao Stephen Hou [2], Han-Jung Lee [3], Melissa H. Cambre [1] and Yue-Wern Huang [1,*]

1 Department of Biological Sciences, Missouri University of Science and Technology, Rolla, MO 65409, USA; larry.tolliver@thermofisher.com (L.M.T.); njhcm8@mst.edu (N.J.H.); mhcxv8@mst.edu (M.H.C.)
2 Department of Biomedical Sciences, University of Wisconsin-Milwaukee, Milwaukee, WI 53211, USA; fyshou@gmail.com
3 Department of Natural Resources and Environmental Studies, National Dong Hwa University, Hualien 97401, Taiwan; hjlee@gms.ndhu.edu.tw
* Correspondence: huangy@mst.edu; Tel.: 1-573-341-6589

Supplementary Table S1: Morphology, APS and SSA of metal oxide nanoparticles [1].

	TiO_2	Cr_2O_3	Mn_2O_3	Fe_2O_3	NiO	CuO	ZnO
SSA (m^2/g)	179	11.4	8.7	31.4	70.9	9	44.6
APS (nm)	46 ± 20	63 ± 34	82 ± 31	48 ± 13	16 ± 5	47 ± 2	27 ± 1
Morphology	Rod-like shape	Roughly spherical	Roughly spherical	Roughly spherical	Roughly spherical	Roughly spherical	Spherical / Rod-like
	From Data Sheet Supplied by Manufacturer						
SSA (m^2/g)	160	N/A	N/A	40	50–80	N/A	50
APS (nm)	50	60	30–60	20–30	10–20	30–50	20

Supplementary Table S2: Cell viability after 24-h exposure to NPs. Each experiment was repeated three times independently with each treatment group having triplicate samples. Data are presented as mean ± standard deviation. Red numbers are significantly different from the control.

	Nanoparticle Concentration (μg/mL)					
Nanoparticle	0	10	25	50	75	100
Cr_2O_3	100.0 ± 0.0	100.7 ± 1.7	95.3 ± 4.8	93.8 ± 1.6	98.6 ± 6.1	98.9 ± 7.0
Fe_2O_3	100.0 ± 0.0	102.2 ± 9.3	100.6 ± 8.0	102.9 ± 7.0	104.4 ± 8.1	106.2 ± 6.5
TiO_2	100.0 ± 0.0	96.3 ± 1.6	81.1 ± 7.0	77.4 ± 3.0	79.1 ± 3.2	76.2 ± 5.1
NiO	100.0 ± 0.0	82.1 ± 0.8	62.4 ± 1.4	55.8 ± 0.8	52.5 ± 2.5	36.8 ± 4.6
Mn_2O_3	100.0 ± 0.0	85.6 ± 4.2	58.1 ± 1.9	45.9 ± 2.0	39.1 ± 7.2	40.4 ± 5.5
	0	4	8	12	16	20
ZnO	100.0 ± 0.0	100.1 ± 3.4	94.0 ± 3.4	73.4 ± 12.8	48.6 ± 2.1	6.8 ± 1.9
CuO	100.0 ± 0.0	24.8 ± 2.6	10.4 ± 2.2	7.7 ± 2.2	5.4 ± 1.6	3.5 ± 1.0

Supplementary Table S3: Cell viability after 48-h exposure to NPs. Each experiment was repeated three times independently with each treatment group having triplicate samples. Data are presented as mean ± standard deviation. Red numbers are significantly different from the control.

Nanoparticle	Nanoparticle Concentration (μg/mL)					
	0	10	25	50	75	100
Cr_2O_3	100.0 ± 0.0	106.1 ± 3.9	101.6 ± 2.4	104.5 ± 6.4	102.7 ± 3.1	104.5 ± 8.0
Fe_2O_3	100.0 ± 0.0	104.4 ± 4.7	100.9 ± 4.8	100.0 ± 3.6	98.3 ± 3.7	92.8 ± 3.0
TiO_2	100.0 ± 0.0	96.8 ± 2.4	84.8 ± 0.6	78.7 ± 1.1	77.7 ± 3.0	72.8 ± 4.7
NiO	100.0 ± 0.0	58.7 ± 3.2	36.6 ± 4.0	30.2 ± 3.0	22.8 ± 2.0	9.7 ± 1.8
Mn_2O_3	100.0 ± 0.0	80.6 ± 6.4	35.2 ± 5.8	20.6 ± 4.5	17.6 ± 5.7	15.7 ± 5.1
	0	4	8	12	16	20
ZnO	100.0 ± 0.0	92.2 ± 5.3	48.4 ± 3.4	4.4 ± 3.2	1.6 ± 0.1	1.6 ± 0.6
CuO	100.0 ± 0.0	30.6 ± 6.8	15.5 ± 4.1	11.8 ± 5.8	8.8 ± 4.4	6.6 ± 2.9

Supplementary Table S4: Apoptosis after 24-h exposure to NPs. Each experiment was repeated three times independently. Data are presented as mean ± standard deviation. Red numbers are significantly different from the control.

Nanoparticle	Nanoparticle Concentration (μg/mL)			
	0	25	50	100
Cr_2O_3	6.9 ± 1.4	7.2 ± 1.4	6.7 ± 0.8	5.8 ± 0.6
Fe_2O_3	4.5 ± 1.4	4.6 ± 1.7	4.9 ± 1.6	5.8 ± 2.1
TiO_2	4.6 ± 1.7	6.3 ± 2.4	9.0 ± 2.3	12.5 ± 2.9
NiO	4.7 ± 2.0	7.4 ± 2.6	7.8 ± 1.2	13.8 ± 2.5
Mn_2O_3	4.6 ± 0.4	9.9 ± 3.2	17.3 ± 4.6	21.6 ± 5.4
	0	5	10	20
ZnO	5.8 ± 2.6	5.1 ± 2.0	10.1 ± 2.1	77.5 ± 7.8
CuO	10.6 ± 2.4	70.2 ± 2.7	79.9 ± 1.5	88.2 ± 5.3

Supplementary Table S5: Apoptosis after 48-h exposure to NPs. Each experiment was repeated three times independently. Data are presented as mean ± standard deviation. Red numbers are significantly different from the control.

Nanoparticle	Nanoparticle Concentration (μg/mL)			
	0	25	50	100
Cr_2O_3	5.1 ± 1.4	4.9 ± 0.2	5.6 ± 0.9	3.5 ± 0.1
Fe_2O_3	4.8 ± 0.6	5.0 ± 0.9	5.3 ± 1.0	6.2 ± 1.7
TiO_2	3.0 ± 0.5	7.3 ± 2.0	7.1 ± 1.0	8.3 ± 0.2
NiO	1.9 ± 0.8	4.2 ± 0.6	6.6 ± 0.2	7.1 ± 1.1
Mn_2O_3	2.7 ± 0.2	7.5 ± 2.2	13.3 ± 4.0	23.8 ± 7.2
	0	5	10	20
ZnO	3.7 ± 0.8	4.2 ± 1.4	4.3 ± 1.0	68.4 ± 6.3
CuO	4.0 ± 1.4	34.8 ± 9.0	54.6 ± 3.3	86.6 ± 4.6

Supplementary Table S6: Proliferation after 24-h exposure to NPs. Each experiment was repeated four times independently with each treatment group having quadruplicate samples. Data are presented as mean ± standard deviation. Red numbers are significantly different from the control.

Nanoparticle	Nanoparticle Concentration (µg/mL)					
	0	10	25	50	75	100
Cr_2O_3	100.0 ± 0.0	99.1 ± 4.3	96.4 ± 3.4	89.7 ± 1.2	84.5 ± 3.2	74.5 ± 10.4
Fe_2O_3	100.0 ± 0.0	94.0 ± 1.6	91.2 ± 3.2	85.4 ± 2.0	84.8 ± 5.2	79.6 ± 0.2
TiO_2	100.0 ± 0.0	90.8 ± 2.1	84.3 ± 1.7	78.8 ± 6.8	71.5 ± 9.2	69.6 ± 8.9
NiO	100.0 ± 0.0	86.6 ± 3.1	76.8 ± 4.2	63.1 ± 7.5	53.8 ± 4.4	43.8 ± 4.6
Mn_2O_3	100.0 ± 0.0	61.1 ± 13.9	31.2 ± 8.9	24.7 ± 8.5	19.6 ± 6.7	15.2 ± 5.5
	0	4	8	12	16	20
ZnO	100.0 ± 0.0	92.8 ± 5.9	71.3 ± 16.0	48.8 ± 6.1	12.5 ± 5.3	1.7 ± 0.3
CuO	100.0 ± 0.0	10.0 ± 4.9	4.0 ± 1.9	2.4 ± 1.1	1.8 ± 0.9	1.7 ± 0.6

Supplementary Table S7: Proliferation after 48-h exposure to NPs. Each experiment was repeated four times independently with each treatment group having quadruplicate samples. Data are presented as mean ± standard deviation. Red numbers are significantly different from the control.

Nanoparticle	Nanoparticle Concentration (µg/mL)					
	0	10	25	50	75	100
Cr_2O_3	100.0 ± 0.0	96.0 ± 3.0	92.4 ± 4.4	87.9 ± 3.4	86.2 ± 3.3	73.9 ± 5.6
Fe_2O_3	100.0 ± 0.0	96.4 ± 7.3	92.3 ± 5.7	88.1 ± 0.5	83.8 ± 3.6	75.3 ± 0.3
TiO_2	100.0 ± 0.0	83.1 ± 4.4	81.6 ± 1.5	76.8 ± 4.0	66.3 ± 2.4	61.6 ± 2.9
NiO	100.0 ± 0.0	80.5 ± 5.9	63.8 ± 7.5	46.8 ± 10.8	33.5 ± 6.7	21.6 ± 4.9
Mn_2O_3	100.0 ± 0.0	27.4 ± 6.3	16.1 ± 6.0	11.3 ± 3.3	7.8 ± 2.6	5.8 ± 2.2
	0	4	8	12	16	20
ZnO	100.0 ± 0.0	91.9 ± 5.0	77.2 ± 12.8	31.2 ± 14.8	1.9 ± 1.5	1.0 ± 0.7
CuO	100.0 ± 0.0	3.1 ± 1.9	1.3 ± 0.4	0.9 ± 0.1	0.7 ± 0.3	0.6 ± 0.3

Supplementary Table S8: Cell cycle distribution after 24- and 48-h exposure to NPs. Each experiment was repeated three times independently. Data are presented as mean ± standard deviation. Red numbers are significantly different from the control.

Nanoparticle	Phase	Nanoparticle Concentration (µg/mL)			
		0	5	10	20
ZnO 24 h	G_0/G_1	58.4 ± 0.8	58.5 ± 2.3	58.3 ± 1.4	55.3 ± 2.8
	S	35.4 ± 1.0	34.6 ± 1.9	33.8 ± 0.9	44.7 ± 2.8
	G_2/M	6.2 ± 0.7	6.9 ± 0.5	7.8 ± 1.3	0.0 ± 0.0
ZnO 48 h	G_0/G_1	61.6 ± 2.3	60.4 ± 2.0	58.7 ± 1.7	50.6 ± 2.3
	S	31.1 ± 2.4	32.5 ± 0.7	34.7 ± 1.0	48.9 ± 2.9
	G_2/M	7.4 ± 0.4	7.0 ± 1.3	6.6 ± 0.7	0.5 ± 0.7
CuO 24 h	G_0/G_1	58.3 ± 1.2	64.7 ± 5.1	61.1 ± 1.2	54.4 ± 3.9
	S	34.0 ± 0.1	34.9 ± 5.1	38.9 ± 1.2	45.6 ± 3.9
	G_2/M	7.7 ± 1.1	0.4 ± 0.6	0.0 ± 0.0	0.0 ± 0.0
CuO 48 h	G_0/G_1	60.3 ± 0.4	46.8 ± 0.5	44.0 ± 2.3	45.1 ± 0.3
	S	33.2 ± 0.1	52.5 ± 1.5	54.9 ± 0.9	54.9 ± 0.3
	G_2/M	6.6 ± 0.4	0.7 ± 1.0	1.1 ± 1.6	0.0 ± 0.0

Supplementary Figure S1: Cell cycle distributions calculated in FCS Express 6 for cells exposed to (A) 0 and (B) 20 µg/mL of ZnO after 24 h

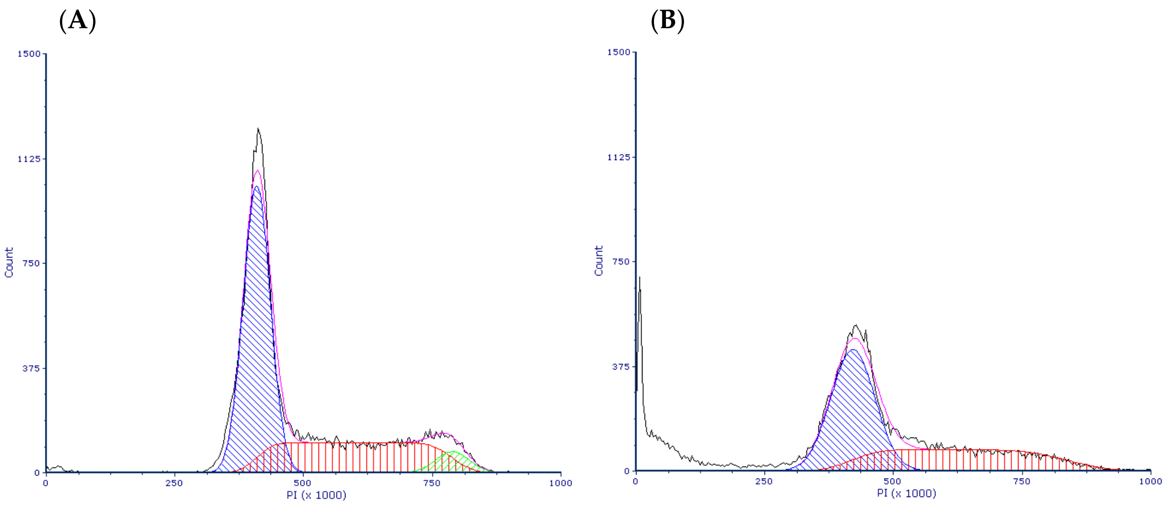

References

1. Chusuei, C.C.; Wu, C.H.; Mallavarapu, S.; Hou, F.Y.; Hsu, C.M.; Winiarz, J.G.; Aronstam, R.S.; Huang, Y.W. Cytotoxicity in the age of nano: the role of fourth period transition metal oxide nanoparticle physicochemical properties. *Chem Biol Interact* **2013**, *206*, 319-326, doi:10.1016/j.cbi.2013.09.020.
